# A Novel Pectin Material: Extraction, Characterization and Gelling Properties

**DOI:** 10.3390/ijms11103686

**Published:** 2010-09-28

**Authors:** Vania Urias-Orona, Agustin Rascón-Chu, Jaime Lizardi-Mendoza, Elizabeth Carvajal-Millán, Alfonso A. Gardea, Benjamín Ramírez-Wong

**Affiliations:** 1 Centro de Investigación en Alimentación y Desarrollo, A.C. Carretera a la Victoria Km 0.6, Hermosillo, Sonora, México; E-Mails: vania@ciad.edu.mx (V.U.-O.); jalim@ciad.mx (J.L.-M.); 2 Centro de Investigación en Alimentación y Desarrollo, A.C. Carretera al Varadero Nacional Km 6.6 Col. Las Playitas 85480 Guaymas, Sonora, México; E-Mail: gardea@ciad.mx; 3 Departamento de Investigación y Posgrado en Alimentos, Universidad de Sonora. Rosales y Transversal 83000. Hermosillo, Sonora, México; E-Mail: bramirez@guaymas.uson.mx

**Keywords:** low methoxy pectin, gels, rheology

## Abstract

A novel pectin was acid extracted from chickpea husk (CHP). CHP presented a 67% (w/w) of galacturonic acid, an intrinsic viscosity of 374 mL/g and a viscosimetric molecular weight of 110 kDa. Fourier transform infrared spectroscopy spectrum of CHP indicated a degree of esterification of about 10%. The CHP-calcium system formed ionic gels with a storage (G′) modulus of 40 Pa and gel set time (G′ > G″) of 3 min at 1% (w/v), and a G′ of 131 Pa and gel set time of 1 min at 2% (w/v). The G′ of CHP gels was not greatly affected by temperature. The results attained suggest that chickpea husk can be a potential source of a gelling pectin material.

## 1. Introduction

Pectin is one of the main structural components of plant cell walls. This polysaccharide is composed of a backbone of (1→4)-linked α-D-galacturonic acid units. The ‘smooth’ homogalacturonic regions are interrupted by ‘hairy’ rhamnogalacturonic regions where galacturonic acid units are interspersed with (1→2)-linked α-L-rhamnopyranosil residues. Rhamnosyl units can be substituted by side chains containing arabinose and galactose. Galacturonic acid residues can be partially esterified by methanol on the carboxyl group and by acetyl on the secondary hydroxyls [1]. Pectin form gels under certain circumstances, the gelling mechanism is highly dependent on the degree of methoxylation (DM). Conventionally, pectin is divided into high methoxy (HM) pectin with DM > 50% and low methoxy (LM) pectin with DM < 50% [2]. Pectin with DM > 50% forms gels in the presence of high sugar concentration, usually sucrose or fructose and low pH; whereas pectin with DM < 50% forms gels in the presence of divalent ions, e.g., calcium, by interaction of carboxyl group ionized (COO^−^) and calcium ions by the “egg box” mechanism [3]. The viscoelastic properties of pectins are the base of their broad use as a gelling agent and stabilizer in food products [2].

Although most plant tissues contain pectin, citrus and apple peel are the major sources of pectic substances around the world [4]. Mexico is the sixth largest exporter of chickpea (*Cicer arietinum* L.) but local consumption is low since chickpea does not represent an important constituent of the Mexican diet. Chickpeas are an excellent source of proteins, fiber, complex carbohydrates, vitamins and minerals. Although most chickpeas are produced for human consumption, low quality grains provide the livestock industry with an alternative protein and energy feedstuff [5]. A large amount of by-products are produced during chickpea processing in regions where this is a major food legume (Southern Europe, North Africa, India and Middle East countries). The majority of chickpea processing wastes include chickpea husk [6], which is used for animal nutrition. A previous research indicated that chickpea husk could be a source of pectinic substances [7]. However, to our knowledge, detailed information on the gelling capability of these polysaccharides in chickpea husk has not been yet reported elsewhere. The aim of this investigation was to extract pectin from chickpea husks (CHP) and to determine its composition, physical-chemical properties and gelling capability.

## 2. Results and Discussion

### 2.1. Pectin Extraction and Characterization

In general, pectin extraction yield from different sources may vary depending on processing parameters (pH, time, temperature) and sample features. Yield of pectin extracted from chickpea husk was 8% (w/w) on a dry matter basis (w pectin/w chickpea husk), which is lower than those reported in major sources of pectic substances like apple fruit (16%) [8]. However, the pectin yield found in the present study is similar to that reported in others husk tissues like cocoa (9%) and sunflower head residues (7–11%) [9,10].

The extracted sample contains 67% (w/w) of galacturonic acid. The main neutral sugars presents in pectin were galactose, arabinose, rhamnose (Table 1). The levels of arabinose and galactose suggest the presence of galactans, arabinans, arabinogalactans and/or rhamnogalacturonan [1]. Other neutral sugar, such as xylose, mannose and glucose were present, but in concentrations below 1.6 g/100 g pectin. The latter could be due to contaminants from other polysaccharides. These results indicate that the polysaccharide was mostly composed of galacturonic acid and a lower proportion of neutral sugars, strongly suggesting that the extracted polysaccharide is pectin. Trace amounts of protein and ash were detected.

Chickpea pectin presented an [*η*] of 374 mL/g, which is similar to that reported in LM pectin from yellow passion fruit and HM pectin from apple [8,11]. The viscosimetric molecular weight (M*v*) of chickpea husk pectin was 110 kDa. This value is in the range reported for pectin extracted from citrus peel (50 to 2000 kDa) [12] and higher to those indicated in LM pectin like citrus and olive pomace pectin (51 and 14 kDa) [13].

**Figure 1:**
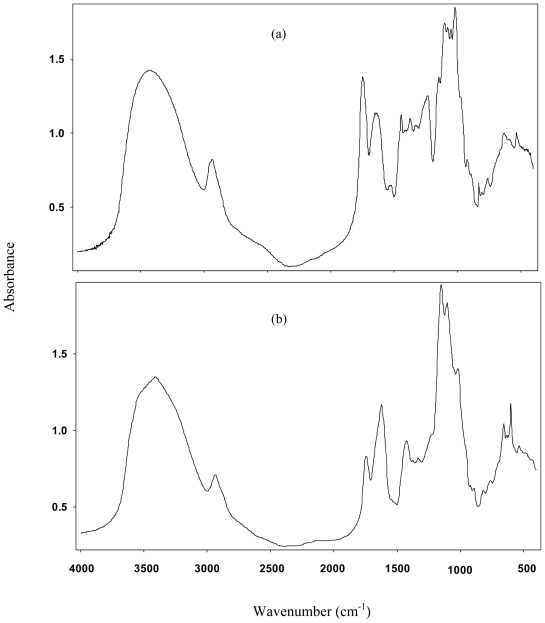
FTIR spectra of apple pectin (**a**) and chickpea husk pectin (**b**).

In order to confirm the identity of chickpea husk pectin extract and estimate the degree of esterification (DE), the sample was analyzed by Fourier Transform Infrared Spectroscopy (FTIR). The CHP spectrum was compared against a commercial pectin standard (Figure 1). It was found that chickpea extract spectra exhibited similarities in its absorption pattern to that of commercial pectin standards. FTIR spectrum in the wavelength range of 950 and 1200 cm^−1^ are considered as the ‘finger print’ region for carbohydrates as it allows the identification of major chemical groups in polysaccharides [14]. Similarities of the husk extract with the pectin standard spectra in the “fingerprint” region suggest that the extract is effectively pectin.

Pectins DE were determined using the peak area relation of the free carboxyl groups (1650 cm^−1^) and esterified groups (1750 cm^−1^) [15]. Pectin esterification degree was calculated by taking the peak areas values of these bands using the following equation:

DE = area of esterified carboxyl groups/(area of esterified carboxyl groups + area of nonesterified carboxyl groups) × 100.

Figure 1 (a), shows the FTIR spectrum of a commercial apple pectin (57% DE), where the absorbance is higher at 1750 cm^−1^ than at 1650 cm^−1^, which is characteristic of a high methoxyl pectin [15]. Figure 1 (b) presents the FTIR spectrum of chickpea husk pectin showing a lower absorbance at 1750 cm^−1^ than at 1650 cm^−1^, indicating a low methoxy pectin. The degree of esterification of chickpea husk pectin was estimated to be about 10%.

### 2.2. Pectin Gelation

**Figure 2:**
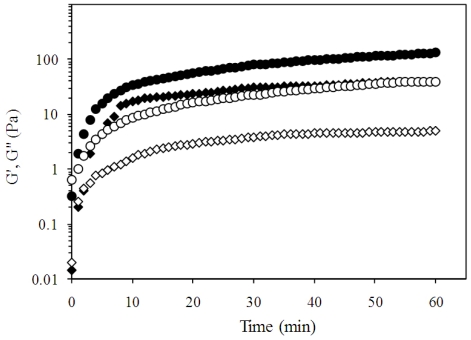
Kinetics of gel formation of chickpea husk pectin at 1 (G′♦, G″ ⋄) and 2 (G′ •, G″ ○)% (w/v) with 10 mmol/L of calcium and pH 5. Measurements at 25 °C, 0.25 Hz and 2.5%. strain.

Gelation of the CHP was studied by dynamic mode rheological analysis. Figure 2 shows the evolution of storage (G′) and loss (G″) modulus as a function of time for a pectin solution at 1 and 2% (w/v). G′ increased rapidly in the first 10 minutes, after which its evolution slows down and turns asymptotic. This behavior is similar to previous report on gelation of LM pectin from olive pomace [16]. By the end of the experiment (60 minutes) G′ and G″ values were 40 and 4 Pa for pectin gels at 1% (w/v) and 131 and 40 Pa for pectin gels at 2% (w/v), respectively, which are higher than those reported for other LM pectin gels [16,17]. In LM pectins, the extent of non-methoxylated galacturonic acid residues is enough for the formation of the so-called ‘egg boxes’. The ‘egg box’ structure is a junction zone resulting of the ionic interaction of the non-methoxylated galacturonic acid blocks that form well adapted cavities where the calcium ions fit in, allowing the linking of two polysaccharide segments [18]. Therefore, the formation of higher amounts of ‘egg box’ structures results in stronger gels. It is possible that the attainment of different G′ values in CHP gels in comparison with other LM pectins could be related to longer galacturonic acid blocks within the extracted CHP chains. During gelation, some other intermolecular interactions like hydrogen bonds could be formed, but they are much weaker as compared to the ionic cross-links formed by carboxyl groups. It has been reported that an increase in branching of pectin results in higher G′ values [19]. CHP branching is low (the ratio of rhamnose to galacturonic acid) but the effect of branching on pectin gelation may be more relevant in HM pectin, as intermolecular association by hydrogen bonding is the primary gelation mechanism. In LM pectin, the interactions between carboxyl groups of pectin and divalent ions are more important for gelation than intermolecular interactions, thus the effect of branching could be reduced [19].

The gel set time of CHP was determined as the time at which G′ and G″ intersected at the study frequency (0.25 Hz). The gel time deduced from rheological measurements corresponded roughly to the moment when a test sample, placed in the same experimental conditions, stopped flowing. Thus, the measurement method (in particular the frequency of measurement) did not significantly affect the gel time, which was found to be three and one minutes for pectin concentrations of 1 and 2% (w/v), respectively.

**Figure 3:**
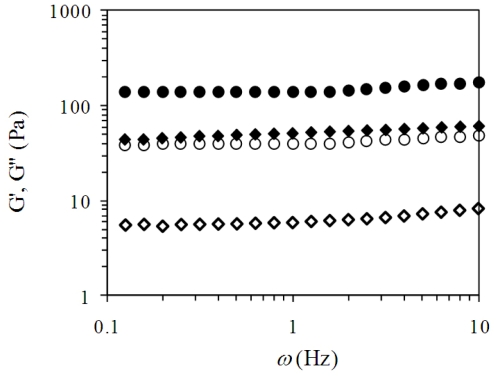
Mechanical spectrum of chickpea husk pectin at 1 (G′♦, G″ ⋄) and 2 (G′ •, G″ ○)% (w/v) with 10 mmol/L of calcium and pH 5. Measurements at 25 °C and 2.5% strain.

Figure 3 shows the mechanical spectrum of CHP after 60 minutes gelation. The mechanical spectrum was typical of solid-like material [20]. G′ was independent of frequency until 1 Hz was reached and increases between 1 and 10 Hz. G″ lineally increases with increasing frequency and was much smaller than G′. This behaviour is similar to that previously reported for a commercial LM pectin (DE 23%) [1]. The tan δ (G″/G′) (data not shown in Figure 3) value calculated at 0.25 Hz was 0.12 and 0.28 for CHP gels at 1 and 2% (w/v), respectively, indicating the presence of an elastic system [21].

**Figure 4:**
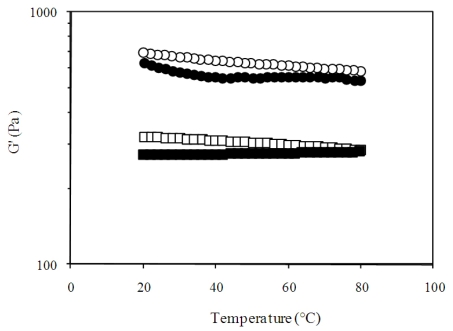
Temperature sweeps (0.25 Hz, 2.5% strain, 1 °C/min) performed for chickpea husk pectin at 1 (heating ▪; cooling □) and 2 (heating •; cooling ○)% (w/v) with 10 mmol/L of calcium and pH 5.

Figure 4 shows the changes in G′ during heating and cooling for cured (20 °C for 18 h) CHP gels at 1 and 2% (w/v). The G′ of CHP gels was not greatly affected by temperature within the temperature range analyzed. The general trend observed is the increase in G′ with decreasing of temperature.

## 3. Experimental Section

### 3.1. Materials

Chickpea (Mocorito-88 variety) samples were kindly provided by the National Institute for Investigation in Forestry, Agriculture and Animal Production in Mexico (INIFAP-CEVACU). All chemical products were purchased from Sigma Chemical Co. (St Louis, MO, USA).

### 3.2. Methods

#### 3.2.1. Pectin Extraction

Chickpea seeds were first heated (1 Kg seeds/2 L water) for 15 min at 50 °C. Husks were then manually separated, dried at 40 °C overnight and milled down to 0.84 mm particle size. Milled husks were dispersed in phosphate buffer (100 g/600 mL) and treated enzymatically for starch and protein degradation, using α-amylase solution (Termamyl®120, pH 7, 100 °C, 30 min, 75 U/g sample), amyloglucosidase, (2 h, 60 °C, pH 5.5, 240 U/g sample, 80 rpm) and pronase (pH 7 at 25 °C to 18 h, followed for 100 °C, 10 min, 0.4 U/g sample). Pectin extraction was performed twice under acid conditions, using 0.05 N HCl (1:6) at 80 °C for 1h and 80 rpm and both supernatants were collected. The extract was centrifuged at 12,040 g for 10 min and the pH adjusted to 3.5. The extract was dispersed into 3 volumes of 96% ethanol during 1 h at 4 °C in order to precipitate pectin, which was then collected by filtration through 4 μm (Whatman) and freeze-dried.

#### 3.2.2. Chemical Composition

Sugar composition was determined according to Carvajal-Millan *et al*. [22] after pectin hydrolysis with 2 N trifluoroacetic acid at 120 °C for 2 h. The reaction was stopped on ice, the extract was evaporated under air at 40 °C and rinsed twice with 200 μL of water and resuspended in 500 μL of water. All samples were filtered through 0.45 μm (Whatman) and analysed by high performance liquid chromatography (HPLC) using a Supelcogel Pb column (300 × 7.8 mm; Supelco, Inc., Bellefont, PA) eluted with 5mM H_2_SO_4_ (filtered 0.2 μm, Whatman) at 0.6 mL/min and 50 °C. A refractive index detector Star 9040 (Varian, St. Helens, Australia) and a Star Chromatography Workstation system control version 5.50 were used. The internal standard was inositol. Ash content was determined according to the AACC methods [23]. Protein was determined by using the Bradford method [24].

#### 3.2.3. Degree of Esterification (DE)

Pectin degree of esterification was determined by FTIR spectroscopy (Nicolet Instrument Corp. Madison, W. I) as described before [15].

#### 3.2.4. Intrinsic Viscosity

Specific viscosity, *η*_sp_ was measured by registering pectin solutions flow time in an Ubbelohde capillary viscometer at 25 ± 0.1 °C, immersed in a temperature controlled bath. Pectin solutions were prepared at different concentrations, dissolving dried pectin in an aqueous solution containing 0.1 N NaCl at pH 7 for 18 h with stirring at room temperature. Pectin solutions and solvent were filtered using 0.45 μm membrane filters before viscosity measurements. The intrinsic viscosity ([*η*]) was estimated from relative viscosity measurements, *η*_rel_, of pectin solutions by extrapolation of Kraemer and Mead and Fouss curves to “zero” concentration [25,26]. NaCl was used in order to prevent pectin aggregation.

#### 3.2.5. Viscosimetric Molecular Weight

The viscosimetric molecular weight (*Mν*) was calculated from the Mark–Houwink relationship, *Mν* = ([*η*]*/k*)^1/^*^α^*, where the constants *k* and *α* are 0.0436 and 0.78, respectively.

#### 3.2.6. Rheological Measurements

The gelling pectin–calcium mixture was prepared at a pectin concentration of 1% (w/v), a calcium content of 10 mmol/L and pH 5. After preparation of the sample, the mixture was quickly transferred onto the rheometer. Rheological tests were performed by small amplitude oscillatory shear by using a strain controlled rheometer (AR-1500ex, TA Instruments, USA.) in oscillatory mode. A plate geometry (5.0 cm in diameter) was used and exposed edges of the sample were covered with mineral oil fluid to prevent evaporation during measurements. Pectin gelation kinetic was monitored at 25 °C for 60 min by following the storage (G′) and loss (G″) modulus. All measurements were carried out at a frequency of 0.25 Hz and 2.5% strain (in linear domain). The mechanical spectra of pectin gels were obtained by frequency sweep from 0.1 to 10 Hz at 2.5% strain and 25 °C. Temperature sweep tests were performed on cured gels (20 °C for 18 h) by heating the samples from 20 to 80 °C, at 1°C/min, and then by cooling from 80 to 20 °C. Measurements were carried out at a frequency of 0.25 Hz and 2.5% strain.

#### 3.2.7. Statistical Analysis

All determinations were made in triplicates and the coefficients of variation were lower than 8%. All results are expressed as mean values.

## 4. Conclusions

The pectin obtained from chickpea husk is a low methoxy pectin capable of forming elastic gels by calcium addition. Gelation kinetics of this pectic/calcium system are in agreement with the general behavior described for other LM pectins. The present research reported the basic properties of this material. The results suggest that chickpea husk can be a potential source of gelling pectin for food and non-food applications. Further research is undergoing in order to explore the structural and functional properties of this polysaccharide.

## Figures and Tables

**Table 1:** Composition of chickpea husk pectin a.

Galacturonic acid	67.0 ± 0.4
Arabinose	7.7 ± 0.3
Galactose	12.3 ± 0.5
Glucose	1.6 ± 0.2
Xylose	0.4 ± 0.1
Mannose	0.6 ± 0.1
Rhamnose	10.4 ± 0.7
Protein	0.02 ± 0.01
Ash	0.03 ± 0.01

a Results are expressed in g/100 g pectin; All results are obtained from triplicates.
